# MAGNET-seq: A tandem PCR and hybrid capture method for enhanced target enrichment

**DOI:** 10.1371/journal.pone.0325385

**Published:** 2025-06-04

**Authors:** Dongin Lee, Taehoon Kim, Duhee Bang

**Affiliations:** Department of Chemistry, Yonsei University, Seoul, Seodaemun-gu, Korea; Peter MacCallum Cancer Institute, AUSTRALIA

## Abstract

Hybrid-capture based target enrichment and multiplex PCR methods enhance sequencing efficiency by focusing on specific genomic regions, while struggling to enrich tens of regions spanning hundreds to thousands of base pairs. We developed MAGNET-seq (Multiplex Amplification and tarGeted eNrichment of sElecTed sequences), a streamlined method that integrates targeted multiplex PCR with hybrid capture. We evaluated its performance using two primer sets: a Drug Resistance Targeting Primers with 43 targets and a Reference Primers set with 7 targets, including clinically relevant mutations such as EGFR c.2369C > T (p.T790M) and KRAS c.35G > T (p.G12C). Using a set of 43 target primers, MAGNET-seq demonstrated higher on-target ratios (average 86.2%) compared to standard targeted multiplex PCR (average 2.2%). Furthermore, MAGNET-seq with 7 target primers showed concordant variant allele frequencies (VAF) in low-VAF (≤ 1%) reference cell-free DNA (cfDNA) samples (0.05% to 1%), supporting its reproducibility. This approach provides a simplified and cost-efficient solution for targeted sequencing, particularly well-suited for applications that require detection of low-allele frequency variants such as somatic cancer mutations.

## Introduction

Targeted sequencing plays a crucial role in genomics research, especially in cancer studies, by providing high depth coverage of specific genomic regions, enabling the detection of somatic variants that may be missed by broader sequencing approaches [1–3]. In cancer research, identifying somatic variants is critical as these mutations are often associated with tumorigenesis [4–6] and may be present at low allelic frequencies, especially in early-stage cancers [7–12] or in heterogeneous tumors [13–15]. Detecting such mutations can inform targeted therapies and enable precision oncology, where treatments are tailored based on the specific genetic alterations within a tumor [2,16–20]. Given the potential for somatic variants to influence treatment outcomes, robust sequencing methods capable of detecting these variants are essential for advancing cancer diagnostics and therapeutics.

However, current enrichment strategies for targeted sequencing, particularly when focusing on tens or hundreds of cancer-associated genomic regions, present inherent limitations. Conventional capture-based target enrichment methods, while typically applied in larger genomic regions, do not effectively obtain the target molecules present in narrow genomic targets spanning hundreds to thousands of base pairs, resulting in low on-target ratios [21,22]. One alternative is amplicon-based target enrichment method, which employs targeted multiplex PCR to simplify the workflow, provided that the target primers are carefully designed [23]. However, this approach faces challenges such as low enrichment efficiency in smaller genomic regions [24,25]. Moreover, increasing the number of targets might risk the formation of primer dimer [26,27], which can reduce the yield of target library molecules [28]. Although several algorithms aim to mitigate primer interactions [29–33], increasing the number of primers makes it challenging to predict primer interactions and necessitates optimization in experimental environments before sequencing [34,35]. Ultimately, both capture-based and amplicon-based approaches remain inefficient for targeted sequencing of narrow genomic regions, highlighting the need for improved methods in these cases.

To overcome these limitations, we developed Multiplex Amplification and tarGeted eNrichment of sElecTed sequences (MAGNET-seq), a novel targeted sequencing library preparation method that combines multiplex PCR with hybrid capture. MAGNET-seq enables tandem target selection, with primary target selection achieved via targeted multiplex PCR, and secondary target selection and artifact removal accomplished through hybrid capture using a whole exome sequencing panel. This approach not only achieves a higher on-target ratio but also improves uniformity in narrow genomic regions compared to standard multiplex PCR, making it particularly effective for detecting low-frequency variants within cancer-associated genes. Moreover, by utilizing custom primers with unique identifier (UID) sequences, PCR errors can be corrected through UID clustering, as demonstrated by Lim et al. in SPIDER-seq (bioRxiv preprint https://doi.org/10.1101/2024.11.26.625438) [36]. Further enhancing its application to cancer research, our method includes two optimized multiplex PCR panels specifically designed to target cancer-related mutations, including those involved in cancer drug resistance to targeted therapies. Finally, MAGNET-seq streamlines the library preparation process by simplifying adapter attachment through consecutive PCR reactions. With these optimized experimental protocols and analysis, MAGNET-seq offers significant potential for cancer genotyping in clinical samples.

## Materials and methods

### Design and screening of PCR primers

The Reference Primers and Drug Resistance Targeting Primers were designed by incorporating adapter sequences, UID sequences, and target-specific primer sequences (S2 Fig). The UID sequence consists of three fixed bases unique to each target primer and a 10-base barcode for distinguishing individual molecules. The primer set targets single nucleotide variants (SNVs), deletions with adjacent targets merged to be covered by a single primer. Primer design was performed using Primer3 (ver. 1.1.4) [37], with target regions padded by 20–80 bp on each side to generate expanded FASTA sequences as input. Primers were filtered using melting temperature (T_m_) criteria of 57.5–62.5°C. Selection prioritized amplicon length minimization to ensure complete coverage of target variants during sequencing, followed by adherence to optimal GC content (40–60%) in both primers of each pair, and minimal positional bias. Positional bias was quantified as half the absolute difference between the distances from the variant to each primer boundary within the amplicon, where a larger value indicates that the variant is located further from the center of the amplicon. Primer specificity was assessed using BLASTN (ver. 2.13.0) against the human reference sequence genome (hg38) [38]. Primers with potential interactions were screened by analyzing pairwise sequence overlaps. A comprehensive list of target regions and the full primer sequences are provided in S1 File.

### MAGNET-seq library preparation

**Fig 1:**
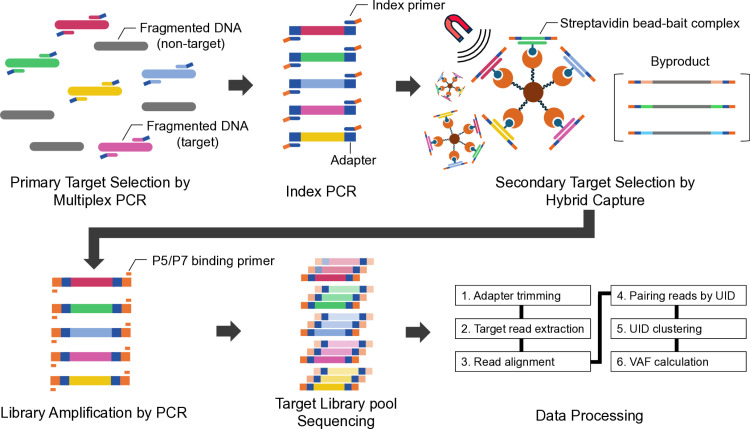
Schematic representation of the MAGNET-seq workflow. In this study, reference cfDNA samples with known variant allele frequencies (VAFs) were used for fragmented DNA. Primary target selection was conducted using targeted primers with flanking adapter sequences, streamlining the process for subsequent amplification with index primers. Secondary target selection was performed via hybrid capture, wherein a streptavidin bead-bait complex was formed to isolate target libraries while removing byproducts through washing, with the complex held by a magnetic field. The final amplification was performed using P5/P7 binding primers to amplify tandem-selected libraries, yielding sufficient quantity for sequencing. The data processing pipeline followed six key steps, as summarized above. A comparison of MAGNET-seq with conventional PCR-based and capture-based target enrichment methods is presented in S1 Fig.

The MAGNET-seq workflow was conducted as follows (Fig 1): First, targeted multiplex PCR was performed using 20 μL of Phusion U Multiplex PCR Master Mix (Thermo Scientific), 4 μL of target primer mix (10 μM), 1 μL of cancer mutation reference sample (10 ng, Seracare), and 15 μL of nuclease-free water, resulting in a total volume of 40 μL. For this study, we used Seraseq® ctDNA MRD Panel Mix (allele frequencies (AF) of 0%, 0.005%, 0.05%, 0.5%) and Seraseq® ctDNA Complete^TM^ Mutation Mix (AF 0.1%, 1%), which contain 22 and 25 cancer-relevant somatic mutations respectively. Throughout this study, we define “low-variant allele frequency (low-VAF) variants” as those present at ≤ 1% VAF, consistent with the Seraseq reference standards analyzed here. Both products are manufactured by spiking biosynthetic DNA variants into cell line-derived DNA backgrounds to create defined AFs, with the DNA processed to mimic the size distribution of cell-free DNA (cfDNA). The mixture was then aliquoted in 8 tubes, with each reaction having a final volume of 5 μL. This step was taken to promote uniform amplification and mitigate potential biases in subsequent index PCR. The PCR cycling conditions were as follows: 98°C for 30 s, followed by 10 cycles of 98°C for 10 s, 60°C for 30 s, and 72°C for 30 s, with a final extension at 72°C for 10 min. For detailed PCR cycling conditions and information on AFs of the samples used, see S2 File.

Second, an index PCR was performed by adding 25 μL of Phusion U Multiplex PCR Master Mix (Thermo Scientific), 2.5 μL of Illumina P5 index primer (10 μM), 2.5 μL of Illumina P7 index primer (10 μM), and 15 μL of nuclease-free water directly to 5 μL of the targeted multiplex PCR product in each tube. To preserve the integrity of the limited starting material and maintain the unique identifiers within the narrow target regions, this step was performed without an intermediate cleanup. Since the same enzyme is used across both PCR steps, and the residual primers are significantly diluted (5 μL in a 50 μL reaction), this approach avoids the loss of UID sequences for low-frequency variants while maintaining efficient amplification. The PCR program was 98°C for 30 s, followed by 10 cycles of 98°C for 10 s, 60°C for 30 s, and 72°C for 30 s, with a final extension at 72°C for 10 min. The PCR products were purified using 1.2X AMPure XP beads (Beckman Coulter).

The index PCR products from the 8 tubes were pooled into a single tube and concentrated using Oligo Clean & Concentrator^TM^ (Zymo Research) to achieve the required input DNA volume for hybrid capture. Hybrid capture was performed using the Celemics Target Enrichment Kit (Celemics) with whole exome sequencing panel, incubated for 8 hours according to manufacturer’s protocol. Dynabeads^TM^ MyOne^TM^ Streptavidin T1 beads (Invitrogen) were applied for magnetic separation of target libraries during hybrid capture. For PCR-only library preparation, the index PCR product was concentrated using the Oligo Clean & Concentrator^TM^ (Zymo Research) to match the equivalent input DNA volume required for the final PCR reaction. A reaction mixture consisting of 50 μL of 2X KAPA master mix, 5 μL of P5 binding primer (10 μM), 5 μL of P7 binding primer (10 μM), 30 μL of streptavidin bead-bait complex, and 10 μL of nuclease-free-water was prepared. The 2X KAPA master mix was prepared using the KAPA HiFi HotStart PCR Kit (KAPA Biosystems) according to the manufacturer’s instructions. The PCR conditions were 98°C for 45 s, followed by 20 cycles of 98°C for 15 s, 60°C for 30 s, and 72°C for 1 min, with a final extension at 72°C for 10 min. The PCR products were purified with 1.2X AMPure XP beads (Beckman Coulter), and quantification of the target libraries was performed using D1000 ScreenTape (Agilent). The pooled libraries were sequenced as 2 × 150 bp paired-end configuration on the NovaSeq 6000 System (Illumina), with an average read count of 60,953,504, ranging from 267,702 to 139,707,648. Detailed information on sequencing metrics for each sample can be found in S2 File.

### Sequencing data processing

Data processing included adapter trimming, target read extraction, read alignment, pairing of reads by UID, UID clustering, and VAF calculation.

Adapter trimming was conducted by FASTP (ver. 0.20.1) [39] with the following parameters:

-q 20 -u 20 -x -y −3 -p -g -t 1 -T 1 –adapter_sequence AGATCGGAAGAGCACACGTCTGAACTCCAGTCAC –adapter_sequence_r2 AGATCGGAAGAGCGTCGTGTAGGGAAAGAGTGTAGATCTCGGTGGTCGCCGTATCATT -w 8.

Target reads containing exact matches with the primer sequences were extracted into separate files. The extracted target reads were then merged into a single file and aligned using BWA (ver. 0.7.17-r1188) [40]. Subsequently, the aligned files were converted to BAM format by SAMTOOLS (ver. 1.7) [41].

During PCR amplification, primers containing UID sequence introduce new UID sequence to daughter strands. However, parent and daughter strands maintain one shared UID, enabling us to track molecular identity. To reconstruct these relationships and reduce PCR-induced errors, read pairs sharing the same UID pair were identified, and those containing soft-clipped reads or with MAPQ < 45 were excluded from further analysis. Clustering was subsequently performed by constructing a peer-to-peer network of UID pairs, where read pairs sharing either the left or right UID were recursively linked to form clusters. This process continued until no additional connections could be made. A cluster thus represents a group of UID pairs that share overlapping UID sequences, reflecting their origin from the same initial DNA molecule.

For confident clustering, each UID pair required a minimum depth of two read pairs, and clusters containing fewer than two unique UID pairs were discarded. Clusters containing more UID pairs than the maximum possible number ($2^{cycle-2}$) were also excluded. The final clusters were assigned a cluster identifier (CID) for downstream analysis, as described by Lim et al. in SPIDER-seq (bioRxiv preprint https://doi.org/10.1101/2024.11.26.625438) [36]. To calculate VAF, a cluster-based consensus approach was used instead of base counts to minimize errors introduced by PCR amplifications. The VAF was calculated using the following formula:


$$VAF=\;\frac{n_{alt}}{n_{alt}\;+n_{ref}\;}$$

where $n_{alt}$ and $n_{ref}$ represent the number of clusters supporting the alternate and reference alleles, respectively. This approach ensures that the calculation is less affected by biases introduced during PCR amplification.

### Analysis of on-target ratio, uniformity, sensitivity, specificity

The on-target ratio was defined as the percentage of total sequenced read pairs that were mapped to the primer target regions. The target-level on-target ratio was calculated as the percentage of total sequenced read pairs mapped to individual target regions, with the overall on-target ratio being the sum of these target-level on-target ratios. The on-target enrichment achieved by MAGNET-seq libraries was determined by dividing the average target-level on-target ratios in MAGNET-seq libraries by the corresponding averages from the PCR-only libraries.

The uniformity calculation was performed using filtered reads that contained exact matches with the targeted primer sequences and were successfully mapped to the corresponding target regions. As each read pair in our dataset can cover an entire targeted region, we determined the total number of filtered read pairs and divided this by the number of target regions to obtain the average number of mapped read pairs per target. Subsequently, we identified the number of target regions with read pair counts falling within 0.5 to 1.5 times the average value [42]. The proportion of target regions with read pairs in this range was considered the uniformity metric.

For VAF analysis of SNV, a depth filtering threshold was applied, defined as the 0.05% of the maximum observed clusters for the respective primer set (Reference Primers set, cutoff ≥ 126). For insertion-deletion (indel) analysis, the sequence overlap between two adjacent deletion variants was calculated by dividing the length of the overlapping region by the total length of the combined target region and sequence. Sequences were assigned to two targets using a sequence overlap cutoff of > 0.9. VAF values for indels were averaged across observations. No filtering based on the number of tubes was applied when analyzing sensitivity and specificity, unlike in VAF comparison analysis, where only variants observed in ≥ 2 tubes were included.

For codon variant specificity, target specificity was evaluated in two steps. First, base-level specificity was assessed by determining whether the percentage of non-reference clusters ≤ 0.1 × the percentage of reference clusters. If this criterion was met, base-level specificity was considered achieved. Second, if at least two bases were identified as specific, target-level specificity was assigned. Details of the variants identified at the codon level are summarized in Table 1.

**Table 1:** Summary of target variants identified by Reference Primers (7).

Gene	Target variant	Variant type	Classified	Primer name	Chromosome	Coordinate
BRAF	BRAF c.1799T > A (p.V600E)	SNV	Positive	RP01	Chr7	140,753,336
EGFR	EGFR c.2235_2249del (p.E746_A750delELREA)	Deletion	Positive	RP02	Chr7	55,174,772−55,174,786
EGFR	EGFR c.2240_2257del (p.L747_P753 > S)	Deletion	Positive	RP02	Chr7	55,174,777−55,174,794
EGFR	EGFR c.2573T > G (p.L858R)	SNV	Positive	RP03	Chr7	55,191,822
EGFR	EGFR c.2369C > T (p.T790M)	SNV	Positive	RP04	Chr7	55,181,378
KRAS	KRAS c.35G > T (p.G12C)	SNV	Positive	RP05	Chr12	25,245,351
KRAS	KRAS c.35G > A (p.G12D)	SNV	Positive	RP05	Chr12	25,245,350
EGFR	EGFR G719X (g.55174014G > A, g.55174014G > C, g.55174014G > T, g.55174015G > A, g.55174015G > C, g.55174015G > T, g.55174016C > A, g.55174016C > G, g.55174016C > T)	Codon	Negative	RP06	Chr7	55,174,014-55,174,016
EGFR	EGFR c.2303G > T (p.S768I)	SNV	Negative	RP07	Chr7	55,181,312

Sensitivity and specificity for individual samples were calculated using the following formulas:


$$Sensitivity=\;\frac{True\;positive\;variants}{True\;positive\;variants+False\;positive\;variants}$$

$$Specificity=\;\frac{True\;negative\;variants}{True\;negative\;variants+False\;negative\;variants}$$

True positive variants were defined as those with observed VAF values ≥ 0.4 × the known VAF values. True negative variants were defined as those where the percentage of non-reference clusters was ≤ 0.1 × the percentage of reference clusters. For the 0% sample, specificity of positive variants was determined by confirming zero mutant allele fraction, while other variants followed the standard threshold for true negatives.

### Statistical analysis

All statistical analyses were performed in R. Figures with p-values were calculated with Wilcoxon rank-sum test.

## Results

### An overview of MAGNET-seq

MAGNET-seq presents a streamlined methodology that integrates targeted multiplex PCR and hybrid capture for highly efficient target enrichment (Fig 1, Materials and Methods). We incorporated UIDs into the targeted primers (Materials and Methods, S2 Fig). These UIDs facilitate the differentiation of sequenced target library molecules [43–45], enabling accurate error correction from PCR-induced variations in priming efficiency during the initial reaction [46–48]. Furthermore, by employing the algorithm proposed by Lim et al., we can cluster UID-matched read pairs. This approach allows us to use higher PCR cycles to ensure robust target library yields, while providing the capability to computationally address potential amplification-related issues.

We designed two sets of target primers: one for drug resistance-associated targets (Drug Resistance Targeting Primers) and another set designed as Reference Primers (S3-S4 Figs). While the Drug Resistance Targeting Primers were designed to cover 43 drug resistance-related targets, the Reference Primers set was designed to target much narrower region, focusing on 7 specific targets. Despite having fewer primers (7 versus 43), the Reference Primers set was strategically developed to complement the analysis by incorporating two deletion variants (Table 1) that were not detected by the Drug Resistance Targeting Primers, providing a more comprehensive evaluation of MAGNET-seq’s capabilities.

### The efficacy of target enrichment in MAGNET-seq is demonstrated through gel electrophoresis and sequenced data

To evaluate the effect of hybrid capture in MAGNET-seq, we prepared two types of libraries: a MAGNET-seq library that underwent both PCR amplification and hybrid capture, and a “PCR-only” library that was subjected to the same PCR amplification but without the subsequent hybrid capture step (Materials and Methods). The primary difference between the PCR-only library and the MAGNET-seq library is the absence of hybrid capture, making the PCR-only library analogous to a conventional targeted multiplex PCR experiment. The PCR-only library serves as a control to demonstrate the advantages of the hybrid capture step in comparison to traditional methods.

**Fig 2:**
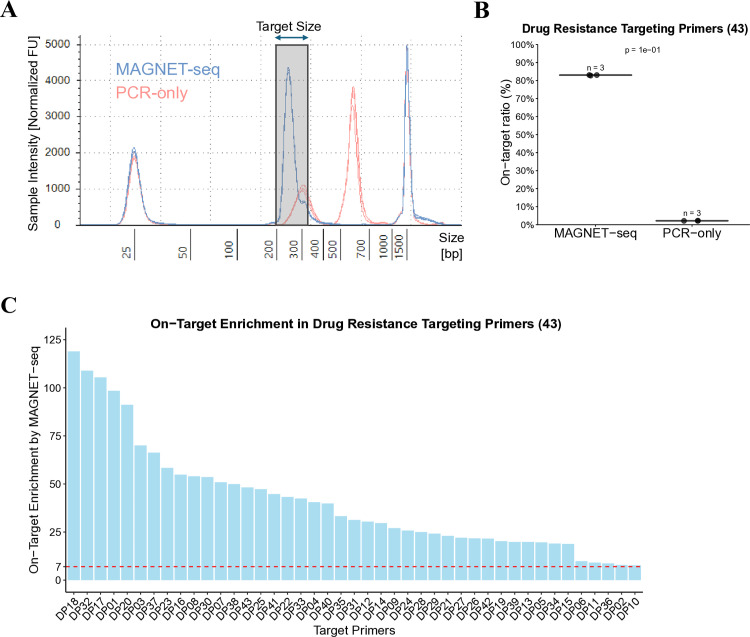
Validation of target Size and on-target ratio in MAGNET-seq. **A** Gel electrophoresis results obtained using the Tapestation D1000 are presented (blue: MAGNET-seq library, red: PCR-only library, n = 3 per group). The gray shaded area indicates the expected target library size range. **B** On-target ratios for the Drug Resistance Targeting Primers are shown. **C** On-target enrichment achieved by MAGNET-seq libraries is shown. The on-target enrichment was determined by calculating the average target-level on-target ratio for each group (MAGNET-seq library, PCR-only library, n = 3 per group). Specific details of the target primers are provided in S1 File.

As shown in Fig 2A, the expected target size range (indicated by gray shading) illustrates the difference in target size peaks between the MAGNET-seq library and the PCR-only library. The expected target library size ranged from 222 to 290 bp, with the average expected target library size estimated to 236.5 bp, which aligns closely with the observed peak in the MAGNET-seq library (S1 File). While the PCR-only library also exhibited a peak corresponding to the expected target size, an additional target peak appeared in 500 and 700 bp, suggesting the presence of potential byproducts in the PCR-only library. These byproducts were absent in the MAGNET-seq library. This result suggests that MAGNET-seq libraries may be more effective in enriching the target library molecules, potentially leading to an increased depth of unique coverage in sequencing data [49–51].

Fig 2B illustrates the on-target ratio of each sample. MAGNET-seq libraries achieved an average on-target ratio of 86.2%, representing approximately a 39-fold enrichment over PCR-only libraries (2.2%). This dramatic increase concentrates sequencing capacity on targeted regions of interest, significantly reducing wasted reads on irrelevant genomic areas while enabling deeper coverage of potential variants at lower overall sequencing costs. Notably, the on-target ratio was significantly higher in MAGNET-seq libraries when using the Reference Primers (S5 Fig), corroborating the results observed with the Drug Resistance Targeting Primers. Furthermore, MAGNET-seq libraries demonstrated superior uniformity across targets compared to PCR-only libraries for both primer sets (S2 File). For instance, MAGNET-seq libraries achieved an average uniformity of 64.3% for the Drug Resistance Targeting Primers, while PCR-only libraries reached 56.6%. Similarly, uniformity with the Reference Primers was notably higher for MAGNET-seq libraries (86.9%) compared to 57.1% in PCR-only libraries.

Upon decomposing the on-target ratio at the individual target level, we found that MAGNET-seq libraries consistently showed enriched data across all targets in the primer set, rather than being disproportionately enriched for a subset of targets (Fig 2C). Here, the enrichment value refers to the fold increase in the proportion of sequenced read pairs mapping to each target region in MAGNET-seq libraries compared to PCR-only libraries (see Materials and Methods). Even the primer showing the lowest enrichment (DP10) from the Drug Resistance Targeting Primer set demonstrated a 7.7-fold increase in target sequence representation. The average enrichment value across all primers was 41.0-fold, underscoring the superior performance of MAGNET-seq libraries in individual target enrichment compared to PCR-only libraries. In conventional targeted multiplex PCR systems, it is common for certain targets to be excluded from analysis due to insufficient depth [52,53], often caused by differential PCR amplification efficiencies among primers [54]. This variability makes it difficult to predict which targets may not be adequately amplified. To address this issue, researchers may consider optimizing primer concentrations by generating initial test data from an equimolar primer mix and adjusting primer concentrations to achieve optimal target enrichment [35]. It is noteworthy that the Drug Resistance Targeting Primers set did not undergo primer concentration optimization for MAGNET-seq libraries, and instead used a mixture of 43 target primers, each present in equivalent molar amounts, thereby reducing the time required for targeted sequencing. Although sufficient sequencing depth was achieved for variant detection in MAGNET-seq libraries, the analysis revealed that target sequences with extreme GC contents (<40% or ≥60%) showed relatively lower on-target ratios (S7 Fig), suggesting that GC content may contribute to the variability in enrichment efficiency across targets.

### Performance evaluation using Reference Primers highlights MAGNET-seq’s reproducibility and potential for practical applications

Using the Reference Primers set described earlier, we thoroughly evaluated MAGNET-seq’s performance across a range of VAFs. An average of 56,457,077 reads were sequenced in 29 samples tested with the Reference Primers set, generating both MAGNET-seq libraries and PCR-only libraries. MAGNET-seq libraries achieved an average of 82.9% on-target ratio, compared to 63.6% in PCR-only libraries, demonstrating superior target enrichment in MAGNET-seq libraries (S5 Fig, S2 File).

**Fig 3:**
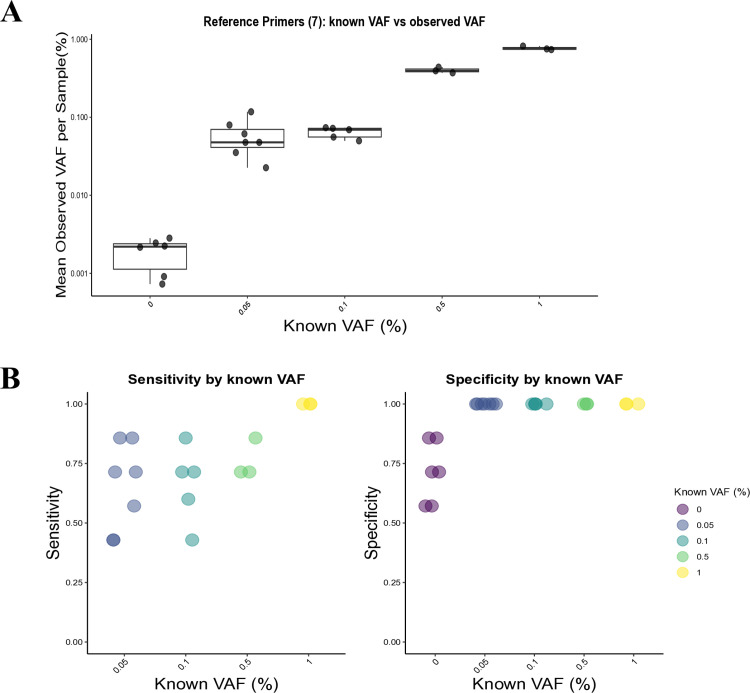
Performance evaluation of MAGNET-seq libraries by Reference Primers. **A** Comparison of known variant allele frequencies (VAFs) with mean observed VAFs per sample. Box plots represent the distribution of observed VAFs for each known VAF level. **B** Sensitivity (left) and specificity (right) of MAGNET-seq libraries across various known VAF levels. Each point represents an individual sample, with colors indicating the known VAF. Sensitivity was evaluated for VAFs between 0.05% to 1%, while specificity was assessed using samples that included 0% VAF.

As illustrated in Fig 3A, the distribution of observed VAF values demonstrates that MAGNET-seq libraries can reliably differentiate samples with a known VAF of 0% and those with VAFs ranging from 0.05% to 1%. The observed VAF distributions for samples with known VAFs of 0%, 0.5%, and 1% were clearly distinguishable from each other. However, two VAF levels (0.05% and 0.1%) showed partially overlapping distributions, positioned between the distributions of 0% and 0.5% VAF samples. The overall results indicate a correlation between the observed VAF values and the known VAFs provided from Seracare. Additionally, the observed VAFs were reproducible across replicates of each reference standard with the same known VAF. Fig 3B further illustrates the performance metrics of MAGNET-seq libraries. The libraries achieved high specificity (1.0) in samples with VAFs between 0.05% to 1% samples. However, a sensitivity of 1.0 was achieved only in samples with a 1% VAF. For all tested VAF levels (0.05%, 0.1%, 0.5%, and 1%), sensitivity values ranged from 0.43 to 1.0, with an average of 0.73. Although further improvements in sensitivity are necessary for samples with VAFs below 1%, these results suggest that MAGNET-seq is a viable method for detecting variants with VAFs of 1% and higher. Detailed analysis of detection rates by variants is in S6 Fig, which shows high indel detection capability.

## Discussion

Conventional capture-based target enrichment approaches offer distinct advantages in cancer genotyping by providing high sequencing depth in specific regions, thus reducing the overall sequencing requirements per sample. However, this method involves several steps for adapter sequence attachment. To address the limitations of the conventional approach, MAGNET-seq streamlines the target enrichment process by incorporating targeted multiplex PCR into the adapter attachment step, reducing it to two PCR reactions. Furthermore, MAGNET-seq employs tandem target selection through hybrid capture, minimizing unwanted amplifications, such as non-target amplicons [55]. This method achieves high efficiency in targeted sequencing by producing a substantial yield of target amplicon libraries, which are subsequently enriched across all targets within the primer set. The use of a standardized whole exome panel for hybrid capture enables robust target enrichment without additional optimization, allowing flexible modification of target regions through primer set selection. The inclusion of UIDs in the primer design further allows for distinguishing PCR-induced errors while maintaining a high number of PCR cycles.

The targets included in our primer set, are associated with drug resistance in targeted cancer therapies. Notably, two key target variants, EGFR c.2369C > T and KRAS c.35G > T, are of clinical relevance for lung cancer [56–61]. This primer set can also be applied for research purposes, as it effectively captures genetic alterations linked to drug resistance, which may be valuable for patient monitoring studies [62–64]. The observed VAF values in low-VAF samples indicate that MAGNET-seq libraries can distinguish samples with low-VAF (Fig 3A); however, the variability in sensitivity across low-VAF samples (Fig 3B, left) highlights the need for further optimization to ensure stable performance.

One key factor affecting sensitivity is the initial DNA input amount (S8 Fig). With only 10 ng of input DNA (approximately 2,900 genomic DNA copies), efficient capture and amplification of each molecule is critical for proper UID cluster formation. During initial PCR, multiple copies with different UID combinations are generated, but when UID loss occurs during capture, original clusters can be fragmented, leading to the failure to meet cluster formation criteria. Since mutant molecules are present in very low numbers (at 0.05% VAF, corresponding to approximately one mutant copy), loss of even a single cluster has a dramatic impact on the analysis, while wild-type molecules maintain sufficient detectable clusters due to their abundance. This limitation produces lower observed VAF compared to predicted values, particularly affecting mutation detection sensitivity. In clinical settings, especially for cfDNA-based diagnostics, obtaining more than 10 ng can be challenging, which makes this technical limitation particularly relevant. Future optimization efforts should focus on both DNA input requirements and PCR conditions to enhance detection while maintaining the method’s streamlined workflow.

MAGNET-seq relies on quality-controlled primer sequences for reliable application in research. Although several widely used tools exist for primer design [37,65–68], inspecting primer sequences to fit specific experimental conditions remains a time- and labor-intensive process [69]. For the Drug Resistance Targeting Primers used in this study, we screened for potential sources of primer-dimers prior to sequencing. For widespread adoption and scalability, further efforts are required to streamline and automate the primer design and screening process, as the current reliance on manual quality control poses a significant bottleneck in time and resources.

Comparative analysis shows that MAGNET-seq lies strategically between low-multiplexing droplet digital PCR (ddPCR), which provides superior quantitative sensitivity for minimal residual disease (MRD) monitoring at VAFs < 0.01% across limited loci, and high-throughput amplicon-based next-generation sequencing (NGS) panels that offer broader mutation coverage with operational simplicity (S1 Table). MAGNET-seq simultaneously interrogates ≥43 loci across multiple samples per run while incorporating UID-based error suppression for reliable variant detection at 0.05–1% VAF. The method’s distinctive advantage lies in its tolerance for highly fragmented DNA templates as short as 60 bp, a capability that challenges conventional amplicon approaches, while maintaining coverage uniformity. This capture-based design enables flexible primer set customization while preserving analytical performance, making MAGNET-seq particularly suitable for comprehensive mutation profiling and longitudinal resistance monitoring where adaptable genomic coverage provides advantages over both alternative methodologies.

Despite its advantages, the architecture of MAGNET-seq introduces practical constraints relative to alternative methodologies. The hybrid capture step, fundamental to MAGNET-seq’s design, extends the workflow by adding 4–72 hours of hybridization incubation time to the two PCR reactions [70–73]. This additional processing time extends the total workflow duration beyond that of amplicon-based NGS panels, making same-day reporting challenging for clinical laboratories. Library preparation costs for MAGNET-seq fall in the mid-range compared to alternatives (S1 Table) but become less economical when monitoring only one or two hotspots, suggesting the method is better suited for multi-sample applications requiring broader coverage. Although MAGNET-seq’s hybrid capture improves coverage uniformity, some genomic regions may still exhibit primer efficiency variations, necessitating brief pilot runs when introducing new panels. Ongoing optimization of capture chemistry and automation should eventually streamline MAGNET-seq for routine clinical implementation, yet its current iteration presents a trade-off between enhanced genomic flexibility and increased procedural complexity compared to simpler amplicon-based approaches.

## Conclusions

Our analysis demonstrates that MAGNET-seq achieves superior target enrichment efficiency with high on-target ratios compared to standard multiplex PCR method, while maintaining reliable detection capabilities across various allele frequencies. The method’s ability to detect variants at low-VAF and its high specificity in detecting drug resistance-associated mutations support its potential utility in cancer genotyping applications. However, the observed variability in sensitivity across low-VAF samples indicates areas for further optimization, particularly in primer design automation and hybrid capture duration. The current challenges in primer screening and extended hybridization times present opportunities for technological improvement, especially for meeting the rapid processing demands of clinical settings. While our validation was performed using ctDNA-like reference samples, the underlying multiplex PCR-based methodology suggests that MAGNET-seq could be adapted for various DNA input types. However, each sample type presents unique challenges: clinical blood samples may exhibit variability in fragmentation patterns across individuals, which could affect on-target ratio and uniformity. In contrast, formalin-fixed, paraffin-embedded (FFPE) samples, while structurally closer to gDNA, might require careful consideration of formalin-induced modifications. Future research should focus on optimizing these processes, validating the method’s performance across diverse sample types, and expanding its practical applications. We anticipate that MAGNET-seq will be applied in future research, contributing to advancements in cancer genotyping and patient monitoring.

## Supporting information

S1 Fig Comparison of target enrichment methods in NGS library preparation. S1 Fig illustrates the workflow of three different types of target enrichment methods: PCR-based, capture-based, and MAGNET-seq. MAGNET-seq integrates tandem target selection by combining targeted PCR and hybrid capture, providing high coverage for selected targets.
(PDF)

S2 Fig Design of the primers and resulting library structures. (A) S2A Fig depicts the structural composition of the designed primer, including the integration of the Unique Identifier (UID). (B) S2B Fig illustrates the library architecture following the attachment of adapter sequences and indexing tags.
(PDF)

S3 Fig UID clustering workflow. To track the UID combinations generated across PCR cycles and integrate them into consensus reads, mapped read pairs are first grouped by UID pairs (each color denotes a unique UID sequence). UID pairs sharing either the left or right UID are then linked recursively via a depth-first search to form UID clusters. Within each cluster, the nucleotide that accounts for ≥50% of all bases is designated the representative base. Finally, variant allele frequency (VAF) is calculated as the number of UID clusters whose representative base matches the alternate allele divided by the total number of UID clusters.
(PDF)

S4 Fig Chromosomal mapping and primer design workflow for Drug Resistance Targeting and Reference Primers. (A) Genomic distribution of Drug Resistance Targeting Primers (red) and Reference Primers (light blue) across human chromosomes. The horizontal bars represent chromosomes 1 through X, with the x-axis indicating chromosome length in megabase pairs (Mbp). Colored vertical lines indicate the genomic coordinates for each primer set. (B) Schematic overview of the primer design and selection process for Drug Resistance Targeting Primers (left) and Reference Primers (right). For the Drug Resistance Targeting Primers, an initial set of 46 primers was designed. Two primers were excluded due to potential primer interactions (one identified through computational screening of sequence overlaps and another through experimental validation). Additionally, during data analysis, one primer was removed due to multi-mapping issues, resulting in a final set of 43 Drug Resistance Targeting Primers. All seven initially designed Reference Primers were retained in the final set.
(PDF)

S5 Fig Comparison of on-target ratios between MAGNET-seq libraries and PCR-only libraries using Reference Primers. The figure compares the on-target ratios (%) between MAGNET-seq libraries and PCR-only libraries. Each data point represents the on-target ratio for an individual sample, and the box plots illustrate the distribution of on-target ratios for each library type. MAGNET-seq libraries exhibit a significantly higher on-target ratio compared to the PCR-only libraries (p < 0.001, Wilcoxon rank-sum test).
(PDF)

S6 Fig Detection rates of variants Across VAF groups using MAGNET-seq libraries with Reference Primers. Detection rates were compared across VAF groups, with samples classified according to their known VAF values. Detection rates were calculated as the ratio of detected samples to total samples within each VAF group. Two indel variants (EGFR c.2240_2257del and EGFR c.2235_2249del) demonstrated consistently high detection rates (>0.9) across all VAF groups.
(PDF)

S7 Fig Relationship between target Sequence GC Content and on-target ratio in MAGNET-seq libraries with Drug Resistance Targeting Primers. Boxplot analysis showing the distribution of on-target ratios across different GC content ranges of target sequences. The on-target ratio represents the mean value from triplicate experiments. Target sequences were categorized into four groups based on their GC content (GC < 40%, 40% ≤ GC < 50%, 50% ≤ GC < 60%, and GC ≥ 60%).
(PDF)

S8 Fig Effect of DNA input amount on MAGNET-seq sensitivity determined by downsampling analysis. Sensitivity for single nucleotide variant detection across different input DNA amounts (1.25, 2.5, 5, and 10 ng) was evaluated using a downsampling approach. DNA input was controlled by varying the number of tubes used in the analysis. The 10 ng samples utilized all 8 tubes (single iteration), while lower input amounts were assessed by randomly selecting the corresponding number of tubes per sample (1,000 iterations per input amount). For each sample, sensitivity values were averaged across iterations. Results show that sensitivity increases with higher input DNA amounts. While 5 ng input showed comparable sensitivity to 10 ng samples, sensitivity decreased progressively at 2.5 ng and 1.25 ng inputs, indicating a threshold for reliable variant detection.
(PDF)

S1 Table Comparison of MAGNET-seq, digital droplet PCR and amplicon-based NGS panels across key performance metrics for somatic mutation analysis. (PDF)

S1 File Target site details of Drug Resistance Targeting Primers and sequences for Drug Resistance Targeting Primers and Reference Primers. (XLSX)

S2 File Sample information detailing experimental conditions (method, primer set, VAF, PCR cycles) and summary statistics (total read pairs, target-mapped read pairs, on-target ratio, uniformity). (XLSX)
