# Supplementary figures and images for: MAGNET-seq: A tandem PCR and hybrid capture method for enhanced target enrichment
Source: PLoS One. 2025 Jun 4;20(6):e0325385. doi: 10.1371/journal.pone.0325385 (PMC12136444; doi:10.1371/journal.pone.0325385)

**Target Enrichment Methods**

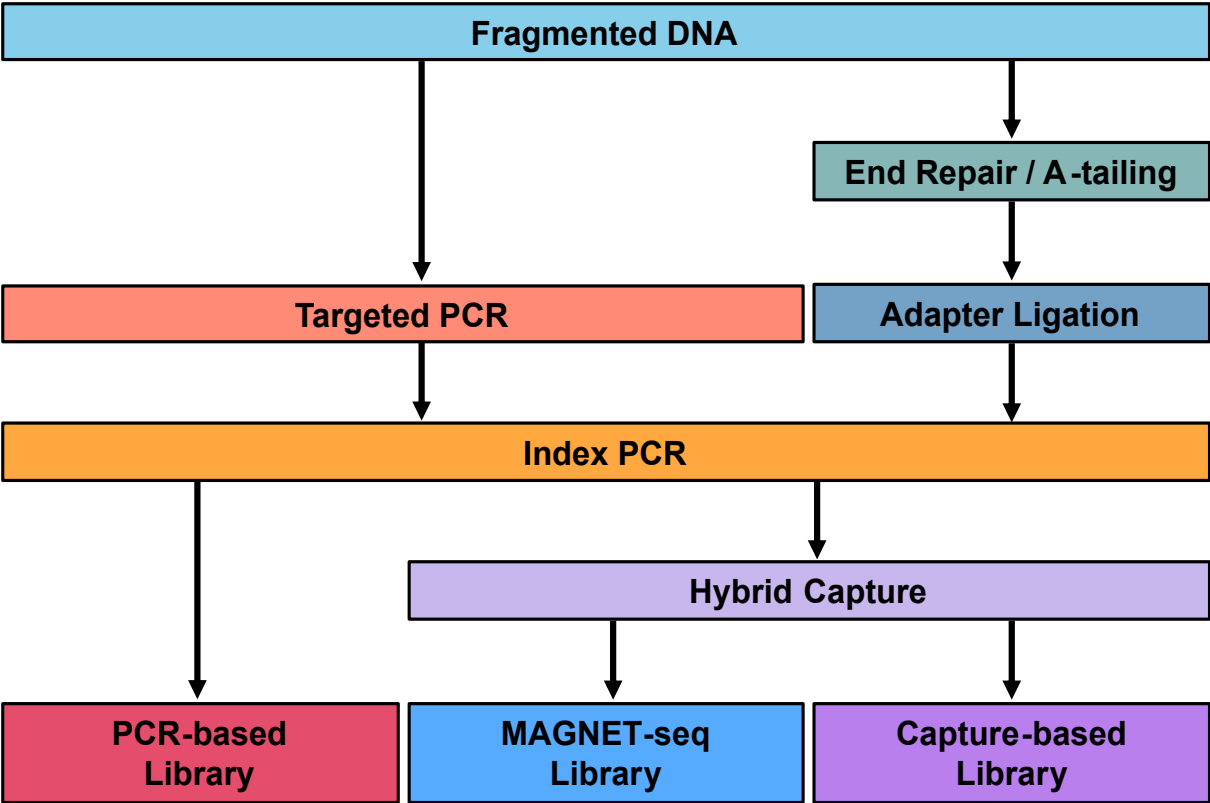

Supplement: S1 Fig — illustrates the workflow of three different types of target enrichment methods: PCR-based, capture-based, and MAGNET-seq. MAGNET-seq integrates tandem target selection by combining targeted PCR and hybrid capture, providing high coverage for selected targets. (PDF) [file pone.0325385.s001.pdf]

Extract UID pairs

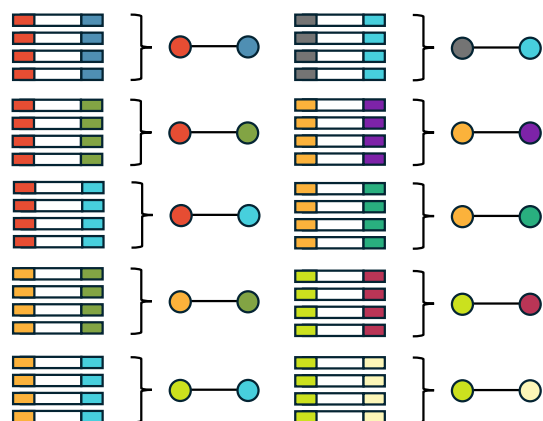

Recursive linking

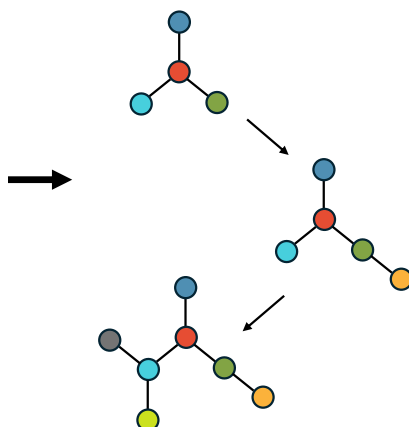

Resulting UID cluster

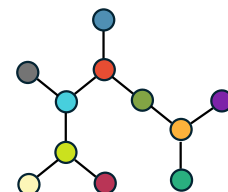

Supplement: S3 Fig — To track the UID combinations generated across PCR cycles and integrate them into consensus reads, mapped read pairs are first grouped by UID pairs (each color denotes a unique UID sequence). UID pairs sharing either the left or right UID are then linked recursively via a depth-first search to form UID clusters. Within each cluster, the nucleotide that accounts for ≥50% of all bases is designated the representative base. Finally, variant allele frequency (VAF) is calculated as the number of UID clusters whose representative base matches the alternate allele divided by the total number of UID clusters. (PDF) [file pone.0325385.s003.pdf]

**A**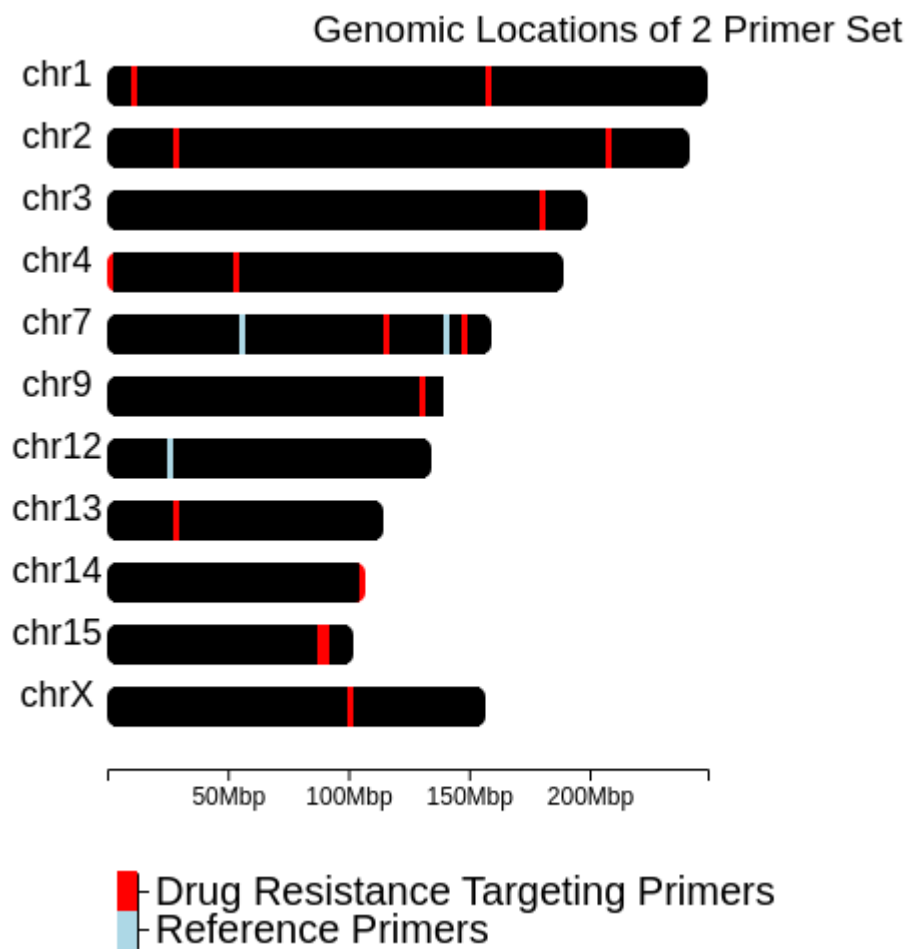**B**

### Drug Resistance Targeting Primers

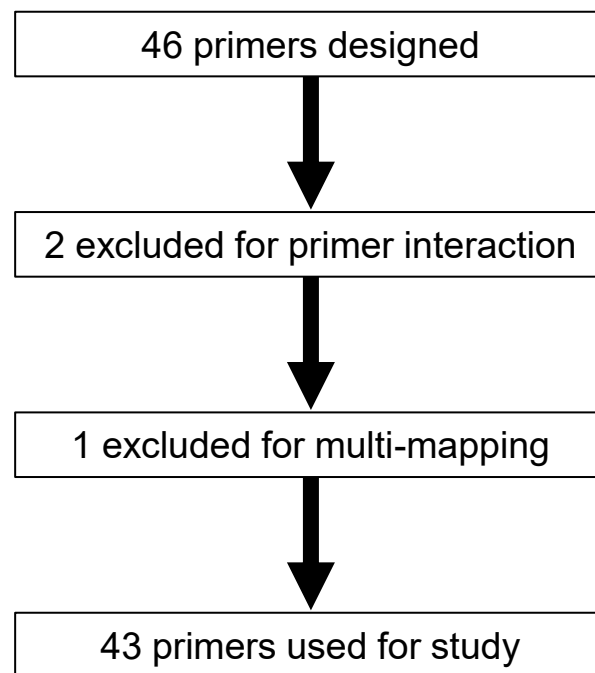

### Reference Primers

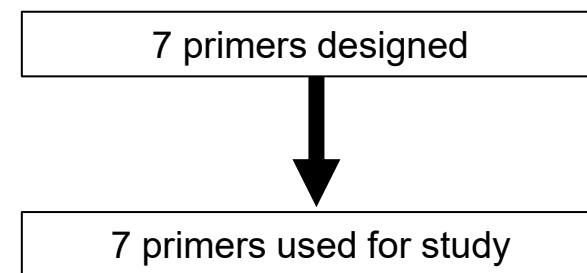

Supplement: S4 Fig — (A) Genomic distribution of Drug Resistance Targeting Primers (red) and Reference Primers (light blue) across human chromosomes. The horizontal bars represent chromosomes 1 through X, with the x-axis indicating chromosome length in megabase pairs (Mbp). Colored vertical lines indicate the genomic coordinates for each primer set. (B) Schematic overview of the primer design and selection process for Drug Resistance Targeting Primers (left) and Reference Primers (right). For the Drug Resistance Targeting Primers, an initial set of 46 primers was designed. Two primers were excluded due to potential primer interactions (one identified through computational screening of sequence overlaps and another through experimental validation). Additionally, during data analysis, one primer was removed due to multi-mapping issues, resulting in a final set of 43 Drug Resistance Targeting Primers. All seven initially designed Reference Primers were retained in the final set. (PDF) [file pone.0325385.s004.pdf]

# Reference Primers (7)

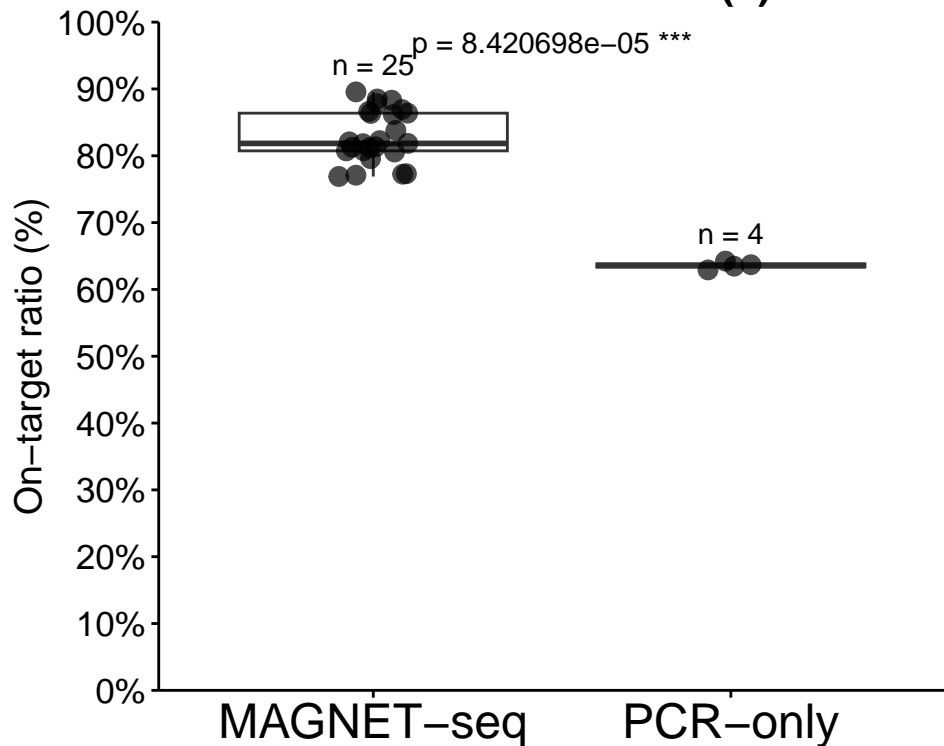

Supplement: S5 Fig — The figure compares the on-target ratios (%) between MAGNET-seq libraries and PCR-only libraries. Each data point represents the on-target ratio for an individual sample, and the box plots illustrate the distribution of on-target ratios for each library type. MAGNET-seq libraries exhibit a significantly higher on-target ratio compared to the PCR-only libraries (p < 0.001, Wilcoxon rank-sum test). (PDF) [file pone.0325385.s005.pdf]

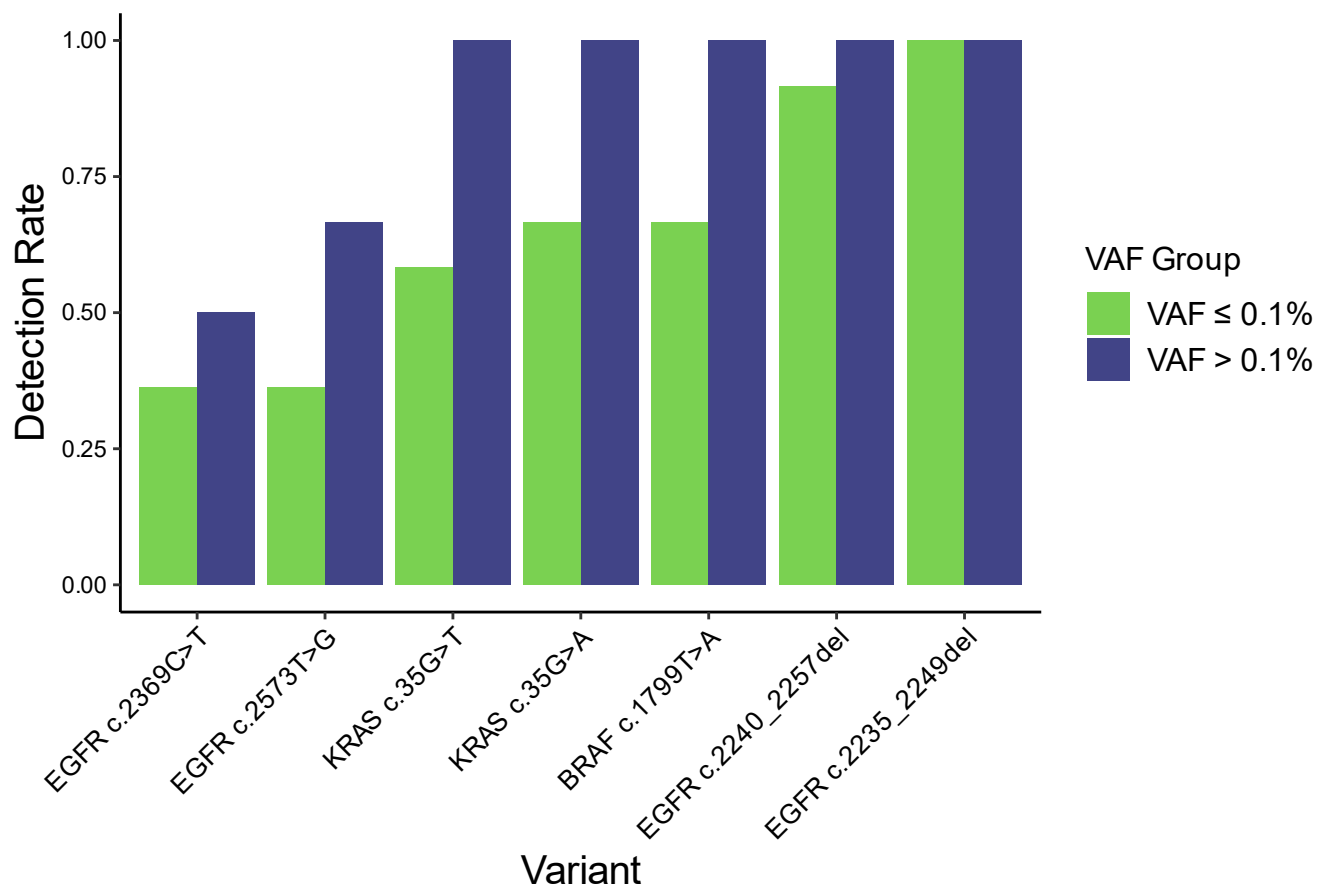

Supplement: S6 Fig — Detection rates were compared across VAF groups, with samples classified according to their known VAF values. Detection rates were calculated as the ratio of detected samples to total samples within each VAF group. Two indel variants (EGFR c.2240_2257del and EGFR c.2235_2249del) demonstrated consistently high detection rates (>0.9) across all VAF groups. (PDF) [file pone.0325385.s006.pdf]

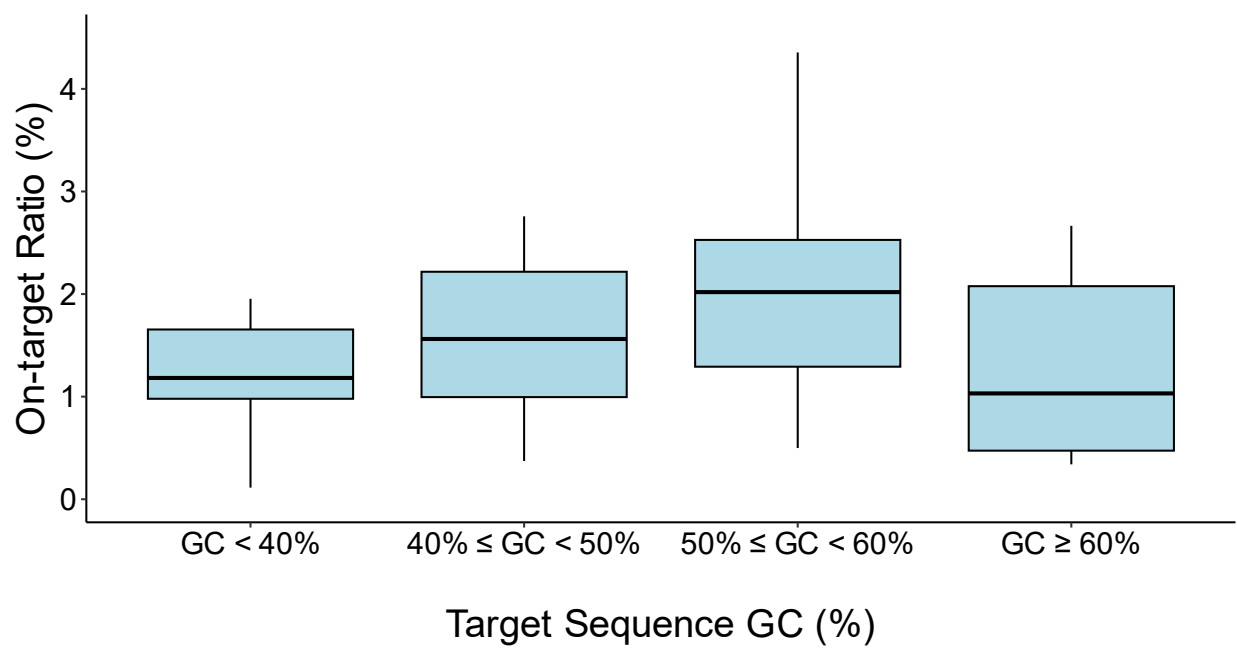

Supplement: S7 Fig — Boxplot analysis showing the distribution of on-target ratios across different GC content ranges of target sequences. The on-target ratio represents the mean value from triplicate experiments. Target sequences were categorized into four groups based on their GC content (GC < 40%, 40% ≤ GC < 50%, 50% ≤ GC < 60%, and GC ≥ 60%). (PDF) [file pone.0325385.s007.pdf]

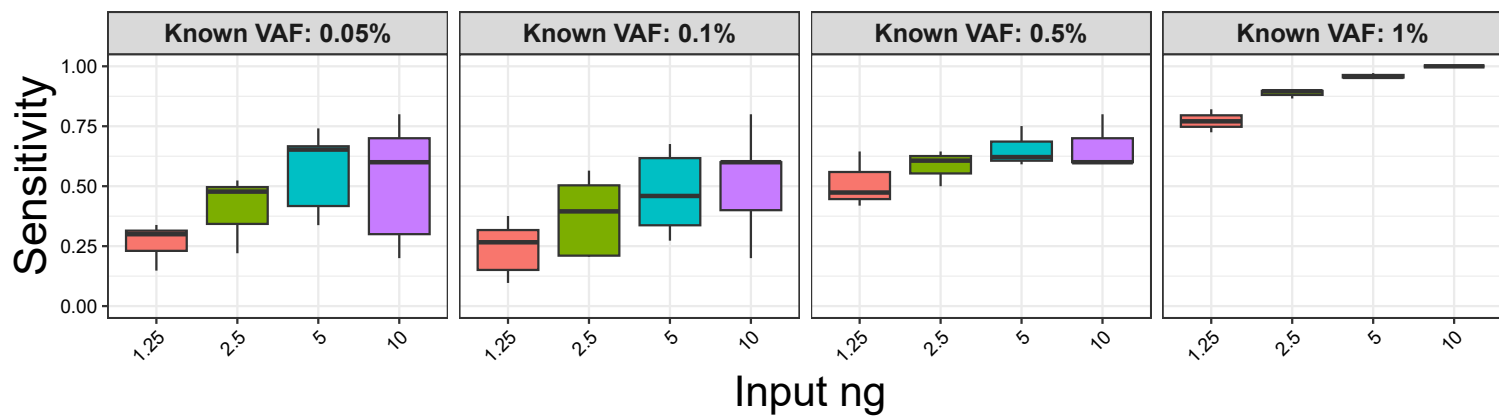

Supplement: S8 Fig — Sensitivity for single nucleotide variant detection across different input DNA amounts (1.25, 2.5, 5, and 10 ng) was evaluated using a downsampling approach. DNA input was controlled by varying the number of tubes used in the analysis. The 10 ng samples utilized all 8 tubes (single iteration), while lower input amounts were assessed by randomly selecting the corresponding number of tubes per sample (1,000 iterations per input amount). For each sample, sensitivity values were averaged across iterations. Results show that sensitivity increases with higher input DNA amounts. While 5 ng input showed comparable sensitivity to 10 ng samples, sensitivity decreased progressively at 2.5 ng and 1.25 ng inputs, indicating a threshold for reliable variant detection. (PDF) [file pone.0325385.s008.pdf]
