# Supplementary material for: MAGNET-seq: A tandem PCR and hybrid capture method for enhanced target enrichment
Source: PLoS One. 2025 Jun 4;20(6):e0325385. doi: 10.1371/journal.pone.0325385 (PMC12136444; doi:10.1371/journal.pone.0325385)
Supplement: S2 Fig — (A) S2A Fig depicts the structural composition of the designed primer, including the integration of the Unique Identifier (UID). (B) S2B Fig illustrates the library architecture following the attachment of adapter sequences and indexing tags. (PDF) [file pone.0325385.s002.pdf]

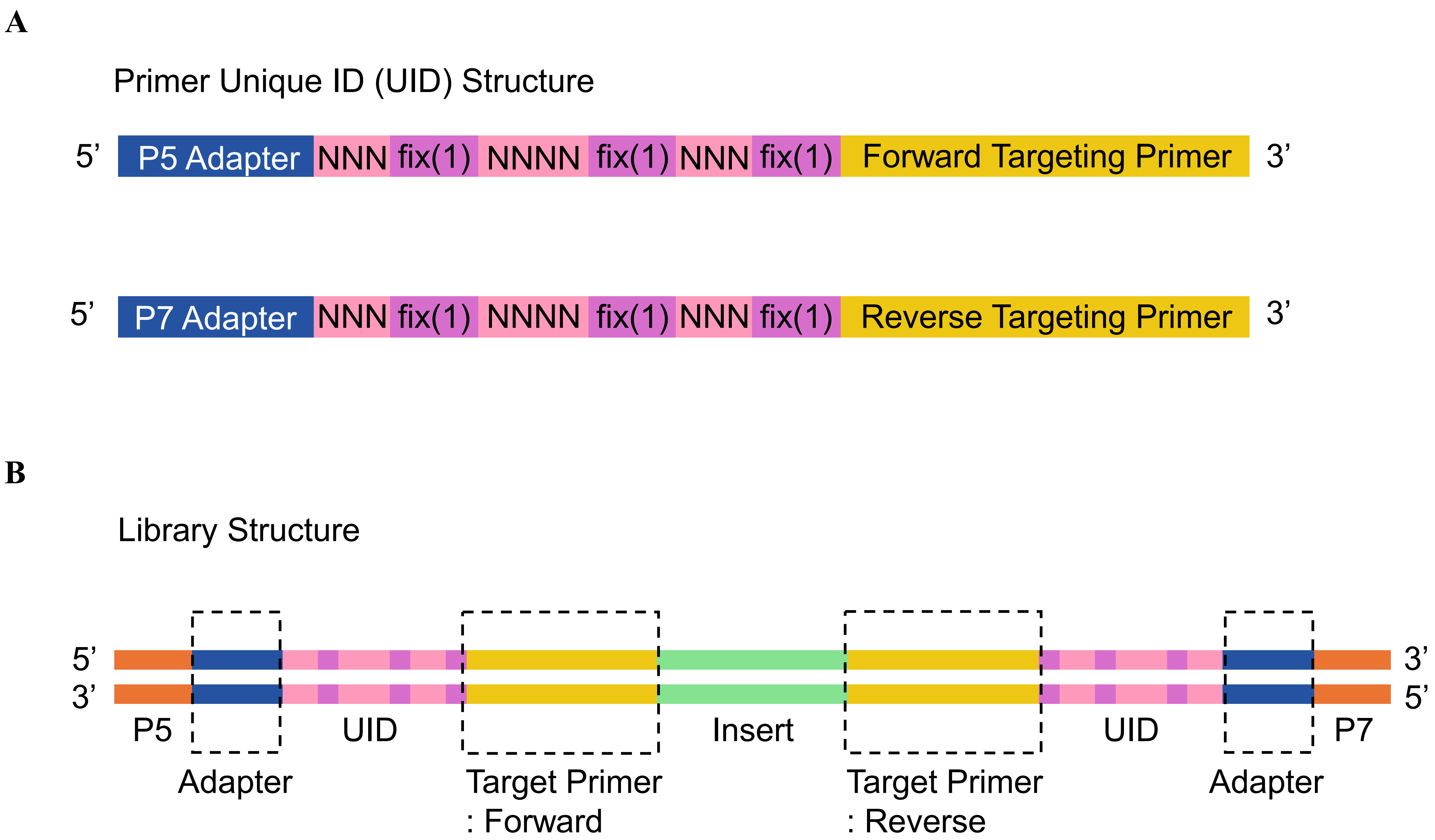
A
Primer Unique ID (UID) Structure
5'
P5 Adapter
NNN fix(1) NNNN fix(1) NNN fix(1)
Forward Targeting Primer
3'
5'
P7 Adapter
NNN fix(1) NNNN fix(1) NNN fix(1)
Reverse Targeting Primer
3'
B
Library Structure
5'
3'
3'
5'
P5
Adapter
UID
Target Primer
: Forward
Insert
Target Primer
: Reverse
UID
Adapter
P7
