# Supplementary material for: MAGNET-seq: A tandem PCR and hybrid capture method for enhanced target enrichment
Source: PLoS One. 2025 Jun 4;20(6):e0325385. doi: 10.1371/journal.pone.0325385 (PMC12136444; doi:10.1371/journal.pone.0325385)
Supplement: S1 Table — (PDF) [file pone.0325385.s009.pdf]

Method	Digital droplet PCR (ddPCR)	Amplicon-based NGS panels	MAGNET-seq
Product	Bio-Rad QX200™ Droplet Digital™ PCR System	Oncomine™ Lung cfDNA Assay (Thermo Fisher Scientific)	Not applicable
Sensitivity[1] (EGFR L858R)	0.697	0.692	1.0 (for VAF 1%)
VAF detection limits	0.001%	0.1%	1%[2]
Sample multiplexing	8	8	≥8
Target multiplexing	2	35 (269: Oncomine Focus)	≥43
Workflow time	2.5 min + 84 min (ddPCR)	46.5 min (PCR)	44 min (PCR) + 8 hr (hybridization)
Cost for library prep (per sample)	$5.0	$326.9 (Oncomine Focus)	$72.8[3]

[1] Sensitivity for the Oncomine panel represents overall EGFR detection rather than the L858R.

[2] Detection limit for MAGNET-seq estimated empirically in this study (10 ng input).

[3] MAGNET-seq cost decreases with sample batching during capture (eight-sample batch shown).

Supporting References

S1. Li C, He Q, Liang H, Cheng B, Li J, Xiong S, et al. Diagnostic accuracy of droplet digital PCR and amplification refractory mutation system PCR for detecting EGFR mutation in cell-free DNA of lung cancer: A meta-analysis. Front Oncol. 2020;10:290. doi:10.3389/fonc.2020.00290

S2. Cho YG, Park J, Han JY, Kim TY. Evaluation of the analytical performance of Oncomine Lung cfDNA assay for detection of plasma EGFR mutations. Genes. 2023;14(6):1219. doi:10.3390/genes14061219

S3. University of Iowa Institute of Human Genetics, Droplet Digital PCR [Internet]. Iowa City (IA): University of Iowa; [cited 2025 Apr 30]. Available from:https://humangenetics.medicine.uiowa.edu/genomics-division/quantitative-dnarna-analysis/droplet-digital-pcr

S4. Kumar S, Bennett A, Campbell PA, Palidwor G, Lo B, Perkins TJ, et al. Costs of next-generation sequencing assays in non-small cell lung cancer: a micro-costing study. Curr Oncol. 2022;29(8):5238–46. doi:10.3390/curroncol29080416.
